# Direct comparison of ten quantitative fecal immunochemical tests for hemoglobin stability in colorectal cancer screening

**DOI:** 10.1038/s41424-018-0035-2

**Published:** 2018-07-06

**Authors:** Anton Gies, Katarina Cuk, Petra Schrotz-King, Hermann Brenner

**Affiliations:** 1 Division of Preventive Oncology, German Cancer Research Center (DKFZ) and National Center for Tumor Diseases (NCT), Heidelberg, Germany; 2 Medical Faculty of Heidelberg, University of Heidelberg, Heidelberg, Germany; 3 Division of Clinical Epidemiology and Aging Research, German Cancer Research Center (DKFZ), Heidelberg, Germany; 4 German Cancer Consortium (DKTK), German Cancer Research Center (DKFZ), Heidelberg, Germany

## Abstract

**Objectives:**

To systematically investigate and directly compare, for the first time, the sample stability of a large number of quantitative fecal immunochemical tests (FITs) at different storage conditions.

**Methods:**

Stool samples were obtained from participants of the German screening colonoscopy program between 2005 and 2010. After an initial FIT-based hemoglobin (Hb) measurement, stool samples were kept frozen at −80 °C until analysis. Twenty randomly selected participants with initial measurements ranging from 10 to 100 μg Hb/g feces were included. Ten quantitative FITs were investigated in parallel. A defined stool amount was extracted using each manufacturer’s brand-specific fecal sampling device and stored at 5 °C, 20 °C, and 35 °C, respectively. After 1, 4, 5, and 7 days, the samples were analyzed blinded. Median fecal Hb concentrations and positivity rates were calculated.

**Results:**

Mean age of the participants was 67 years (range: 56–80 years) and 60% were male. The most advanced finding at screening colposcopy was advanced adenoma in five and non-advanced adenoma in eight cases. Hyperplastic polyps were found in two participants and five participants were without any findings. At 5 °C storage temperature, almost all FITs showed fairly stable results throughout the 7-day observation period. At 20 °C, most FITs still showed fairly stable results over 4 days, whereas positivity rates significantly declined from day 4 on for most FITs at 35 °C. Major differences regarding the sample stability between FITs were observed.

**Conclusion:**

FIT-specific Hb decay according to ambient temperature and time period between sampling and test evaluation requires careful consideration in the design of FIT-based screening programs.

## Introduction

Colorectal cancer (CRC) is the third most common cancer with approximately 1.4 million new cases and 700,000 deaths per year worldwide^1^. Randomized trials have demonstrated that screening with guaiac-based fecal occult blood tests can reduce CRC mortality by up to 30%^2–4^. Even larger effects should be possible with fecal immunochemical tests (FITs) for hemoglobin (Hb), which were shown to enable substantially better diagnostic performance^5–7^ and higher adherence rates in routine screening practice^8,9^. Therefore, FITs are meanwhile widely recommended and used in many countries as primary CRC screening tests^10–12^. Due to the growing market for FIT-based screening, a large number of FITs from diverse manufactures are meanwhile being offered^13^.

Each FIT manufacturer uses a brand-specific fecal sampling device (FSD), which is a small vial containing an Hb-stabilizing buffer and a plastic stick for the collection of a defined amount of stool. In a recent publication from our group, we evaluated and directly compared, for the first time, the diagnostic performance of nine quantitative FITs and found very similar diagnostic performance among all tests, after adjusting the positivity thresholds to yield equal specificities^14^. However, in this study, we evaluated the fecal samples under equal pre-analytical conditions, including identical sample storage temperature and duration. It is therefore unclear, to what extent diagnostic performance may be affected by potential differences of various FITs in Hb stabilization when the tests are applied under routine conditions, where variation in ambient temperature and time from sampling to analysis are the rule rather than the exception.

We therefore aimed to investigate and directly compare Hb decay and its potential impact on positivity rates (PRs) at various temperatures and sample storing times to be expected under routine screening conditions for the same nine quantitative FITs included in the previous examination plus an additional, previously not included quantitative FIT.

## Methods

This article is following the STARD (Standards for Reporting of Diagnostic Accuracy) statement^15^ and the FITTER (Fecal Immunochemical Tests for Hemoglobin Evaluation Reporting) checklist^16^.

### Study design and study population

This project is based on the BliTz (Begleitende Evaluierung innovativer Testverfahren zur Darmkrebsfrüherkennung) study, an ongoing prospective study conducted among participants of screening colonoscopy. The study is carried out in cooperation with 20 gastroenterology practices in Southern Germany with the aim to collect blood and stool samples for evaluating novel non-invasive CRC screening tests. Participants of the German screening colonoscopy program are informed and recruited at a preparatory visit in the practice, typically 1 week before colonoscopy.

Further detailed information on the BliTz study has been provided elsewhere^7,14,17–20^. The study has been approved by the Ethics committees of the University of Heidelberg and of the State Chambers of Physicians of Baden-Wuerttemberg, Rhineland-Palatinate and Hesse.

For this project, we considered study participants who were recruited from 2005 to 2010 and provided stool samples in 60ml-containers (*n* = 2042). After giving written informed consent, the participants were asked to collect one stool sample from a single bowel movement, without any specific recommendations for dietary or medicinal restrictions, before starting the bowel preparation for colonoscopy. Participants were furthermore asked to keep the stool-filled container in a freezer or, if not possible, in a refrigerator at home until their colonoscopy appointment. Upon receipt, the stool-filled containers were kept  at −20 °C in the practice, then shipped on dry ice to a central laboratory and finally stored at −80 °C at the German Cancer Research Center (DKFZ). Median time from stool collection by the study participants to arrival at DKFZ and storage at −80 °C was 9 days (interquartile range (IQR): 6-14 days) .

After excluding 530 participants who were already evaluated in the aforementioned previous study from our group^14^, 1512 participants were potentially eligible. In order to focus on the range of initial FIT values for which a potential Hb decay might turn a positive result to a negative result is of largest concern, we identified 35 individuals with fecal Hb concentrations between 10 and 100 μg Hb/g feces based on quantitative measurements by one of the FITs (*OC Sensor*) in a previous analysis^7,20^. Out of these, seven participants had to be excluded due to an insufficient stool amount. Finally, 20 out of 28 eligible samples were randomly selected and included in this stability analysis.

### FITs included and laboratory analyses

Detailed information on ten quantitative FITs is shown in Table 1. Six laboratory-based and four point of care tests were included. Each FIT sampling tube was filled with a brand-specific Hb stabilization buffer to slow down any Hb decay from sampling until test evaluation. All FITs were read automatically, ruling out potential interpretation bias by test readers. One point of care test (QuantOn Hem) did not even require a local analytical instrument, but could be evaluated using a smartphone with an App for optical analysis of the test cassette.

**Table 1:** Overview of ten quantitative FITs

FIT brand	Manufacturer	Fecal sampling device (fecal mass/buffer volume)	Analytical instrument	Analytical range (µg Hb/g feces)	Preset threshold (µg Hb/g feces)
*Laboratory based*					
CAREprime Hb	Alfresa Pharma (Osaka, Japan)	Specimen collection container A (10 mg/1.9 ml)	CAREprime	0.76–228	6.30
ELISA Test Hb	ImmuChrom (Heppenheim, Germany)	Frost Diagnostika stool collecting tube (40 mg/2 ml)	ELx 800 ELISA Reader	0.44–21	2.0
IDK Hb ELISA	Immundiagnostik (Bensheim, Germany)	IDK extract (15 mg/1.5 ml)	Dynex DSX	0.086–50	2.0
OC Sensor	Eiken Chemical (Tokyo, Japan)	OC auto-sampling bottle 3 (10 mg/2.0 ml)	OC Sensor io	10.0–200	10.0
RIDASCREEN Hb	R-Biopharm (Darmstadt, Germany)	RIDA TUBE Hb (10 mg/2.5 ml)	Dynex DSX	0.65–50	8.0
SENTiFIT-FOB Gold	Sentinel Diagnostics (Milan, Italy)	SENTiFIT pierceTube (10 mg/1.7 ml)	SENTiFIT 270 analyzer	1.70–129.88	17.0
*Point of care*					
Eurolyser FOB test	Eurolyser Diagnostica (Salzburg, Austria)	Eurolyser FOB sample Collector (19.9 mg/1.6 ml)	Eurolyser CUBE	2.01–80.4	8.04
immoCARE-C	CARE diagnostica a (Voerde, Germany)	Sample collection tube (20 mg/2.5 ml)	immoCARE Test cassette + CAREcube	3.75–250	6.25
QuantOn Hem	Immundiagnostik (Bensheim, Germany)	QuantOn Hem TUBE (15 mg/1.5 ml)	QuantOn Hem Test cassette + Smartphone App/iOS^a^	0.30–100	3.70
QuikRead go iFOBT	Orion Diagnostica (Espoo, Finland)	QuikRead go iFOBT Sampling Set (10 mg/2.0 ml)	QuikRead go	15–200	15.0

*FIT* fecal immunochemical test, *Hb* Hemoglobin, *App* mobile application software, *iOS* iPhone operating system

^a^iPhone 6 s with special software for test analysis (designed by the manufacturer) was used for this study

**Fig. 1:**
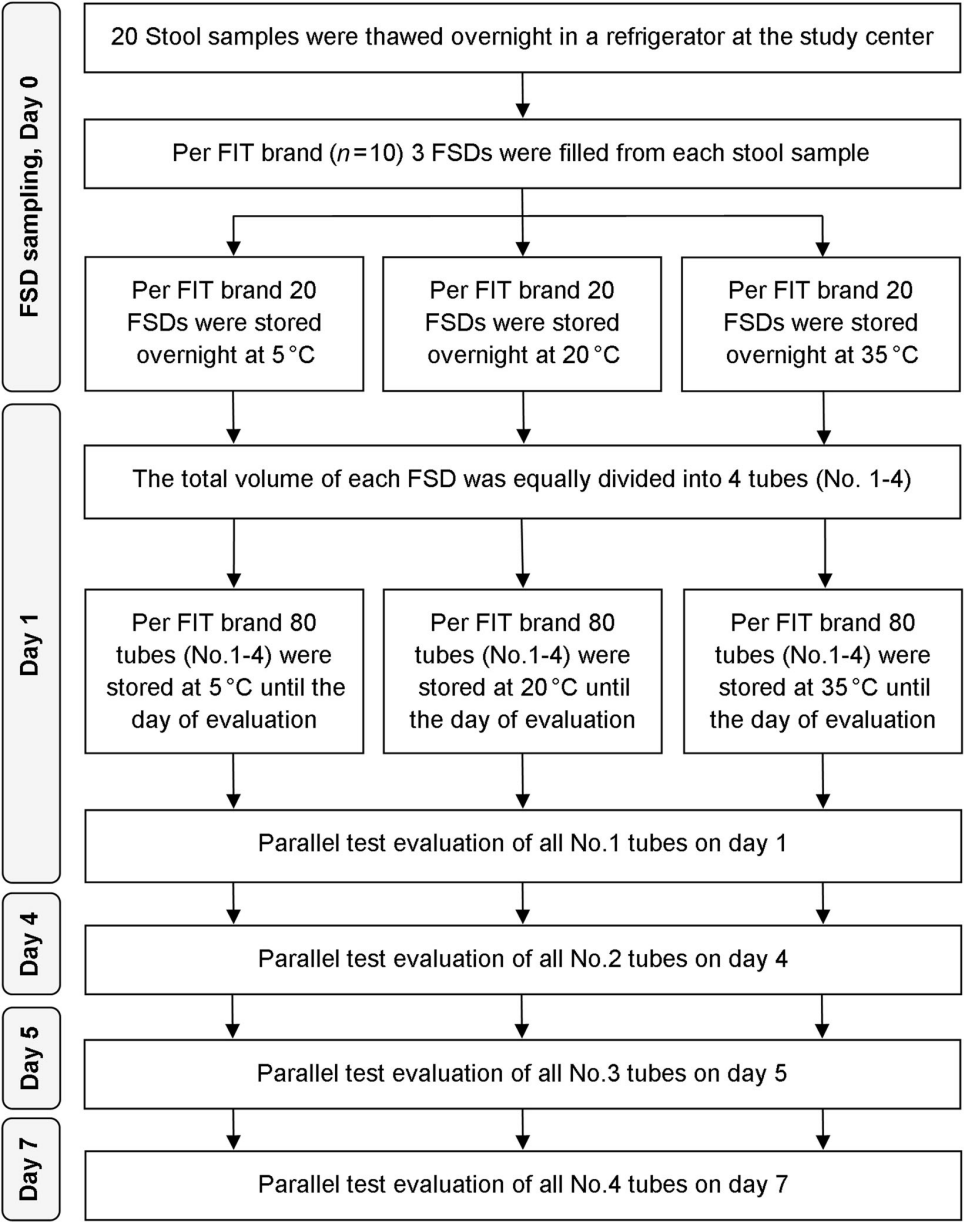
Flow diagram of the workflow in this study. FSD fecal sampling device, FIT fecal immunochemical test

Figure 1 shows the workflow of this study. The stool samples were thawed overnight in a refrigerator at the German Cancer Research Center (DKFZ) and homogenized afterwards. A defined amount of stool was extracted in a randomized order using each manufacturer’s brand-specific FSD. Three FSDs of each manufacturer were filled with each of the stool samples (*n* = 20) and stored at 5, 20, and 35 °C, respectively. Each FSD was a small vial, filled with a defined volume of brand-specific Hb-stabilizing buffer and containing a plastic stick for stool collection. After stabbing the collection stick into three different parts of the fecal sample, it was checked if the collection stick was optimally filled with a sufficient amount of stool. Then the stick with the collected stool was inserted back into the vial. The vials have a tight entrance which removes excess stool, leaving only a defined quantitative amount of stool on the stick. The only exception was the *immoCARE-C* vial, where a supplied custom-fitted scraper was used to remove excess stool from the collection stick. All FSDs were subsequently mixed on a vortexer, so that the stool could disperse into the preservative buffer to ensure optimal Hb stabilization. Afterwards, the respective FSDs were stored overnight at a temperature of 5, 20, and 35 °C, respectively.

On the following day, all FSDs were homogenized in a vortexer, and the total volume of each FSD was equally divided into four 0.5-ml safe-lock tubes. Each of the four tubes was assigned to a single measurement on one of the chosen 4 days of evaluation. All tubes (*n* = 240 for each FIT brand) were labeled with a random number to ensure blinded analysis. After aliquoting, the filled tubes were immediately put back in the respective storage temperature (5, 20, and 35 °C, respectively) until their assigned day of evaluation.

On the specific day of measurement (1, 4, 5, and 7, respectively), only the tubes that were assigned to be measured on that day were taken out to room temperature for test evaluation.

Test analyses of all FIT brands were conducted in parallel by laboratory-experienced staff and test calibrators, as well as test controls were performed on a regular basis according to the manufacturers' instructions. The storage temperatures were automatically recorded every 15 min using the temperature data logger.

Due to limited laboratory space and resources, six tests had to be evaluated externally. On the morning of each of the four measurement days, the test aliquots were packed in a temperature-isolated manner and directly shipped by one logistic company (Gold Key Logistics, Heidelberg, Germany) to the cooperating companies providing the respective tests (CARE diagnostica [*CAREprime Hb* and *immoCARE-C*], Frost Diagnostika [*ELISA Test Hb*], Immundiagnostik [*IDK Hb ELISA* and *QuantOn Hem*], and R-Biopharm [*RIDASCREEN Hb*]) for immediate test evaluation upon arrival on the same day. The tubes which were stored at 5 °C until the evaluation was transported in a cold-chain, whereas the other tubes were transported without any cooling.

### Statistical analyses

**Fig. 2:**
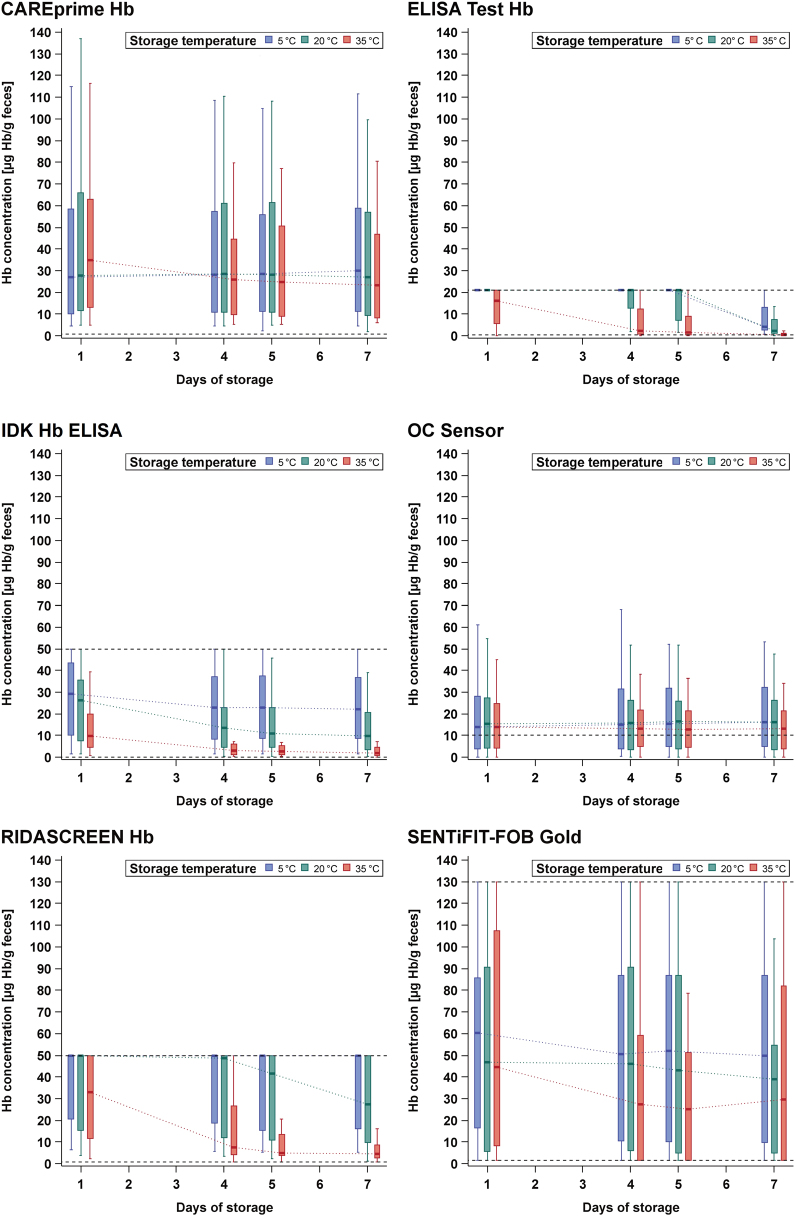

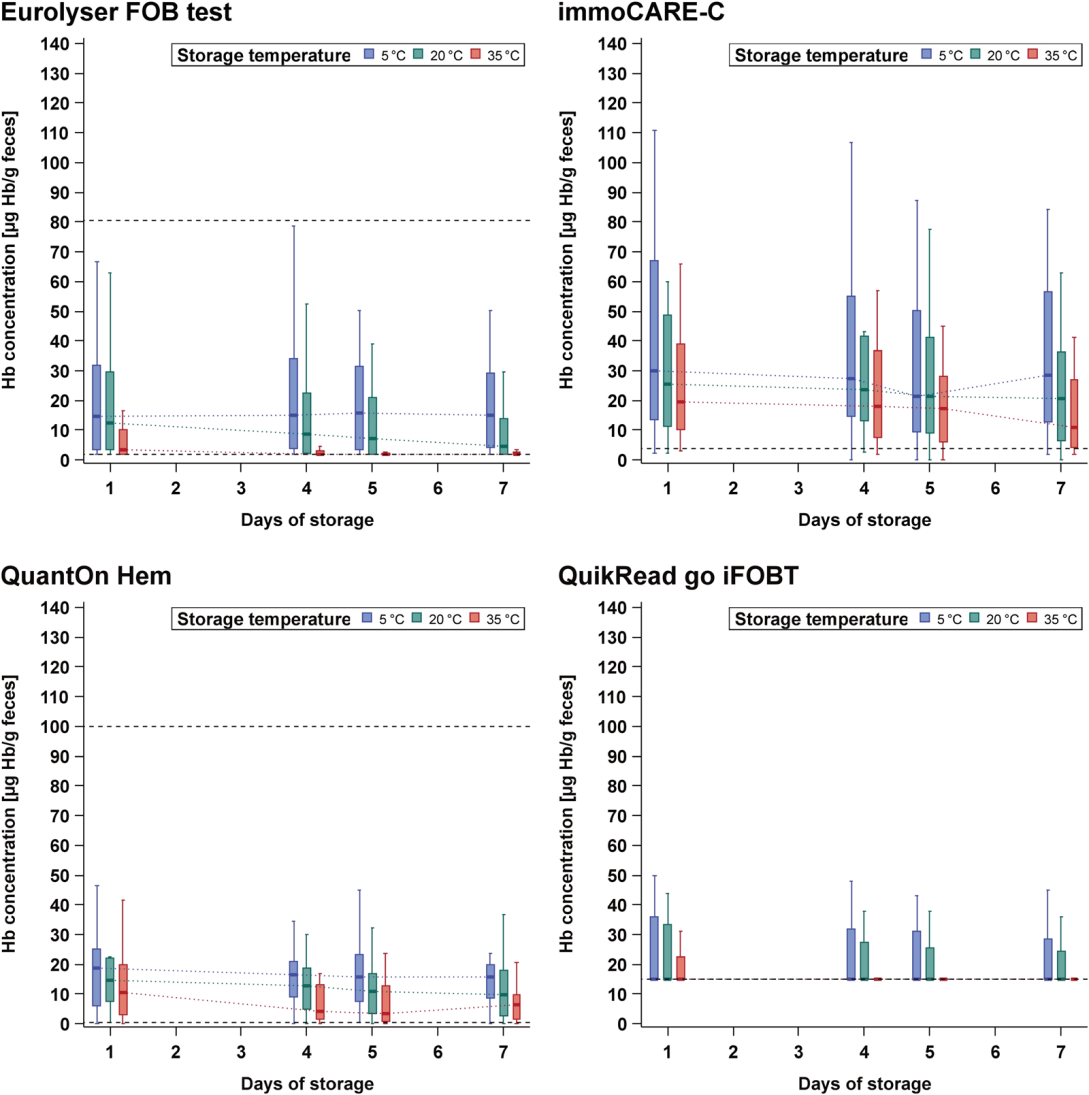
Hemoglobin concentration according to storage conditions. Hb hemoglobin. Black horizontal dashed lines = upper and lower analytical limit. *The analysis of CAREprime is based on 19 samples on the seventh day at 20 °C storage, whereas all other analyses are based on all 20 samples

The median fecal Hb concentrations with their IQR and whiskers within 1.5 IQR were calculated and displayed according to the different lengths of storage at different temperatures (Fig. 2). In addition, differences in fecal Hb concentrations between various combinations of storage conditions (temperature and time) and 1-day storage at 5 °C were evaluated using Wilcoxon signed-rank test (Supplementary Table 2).

**Fig. 3:**
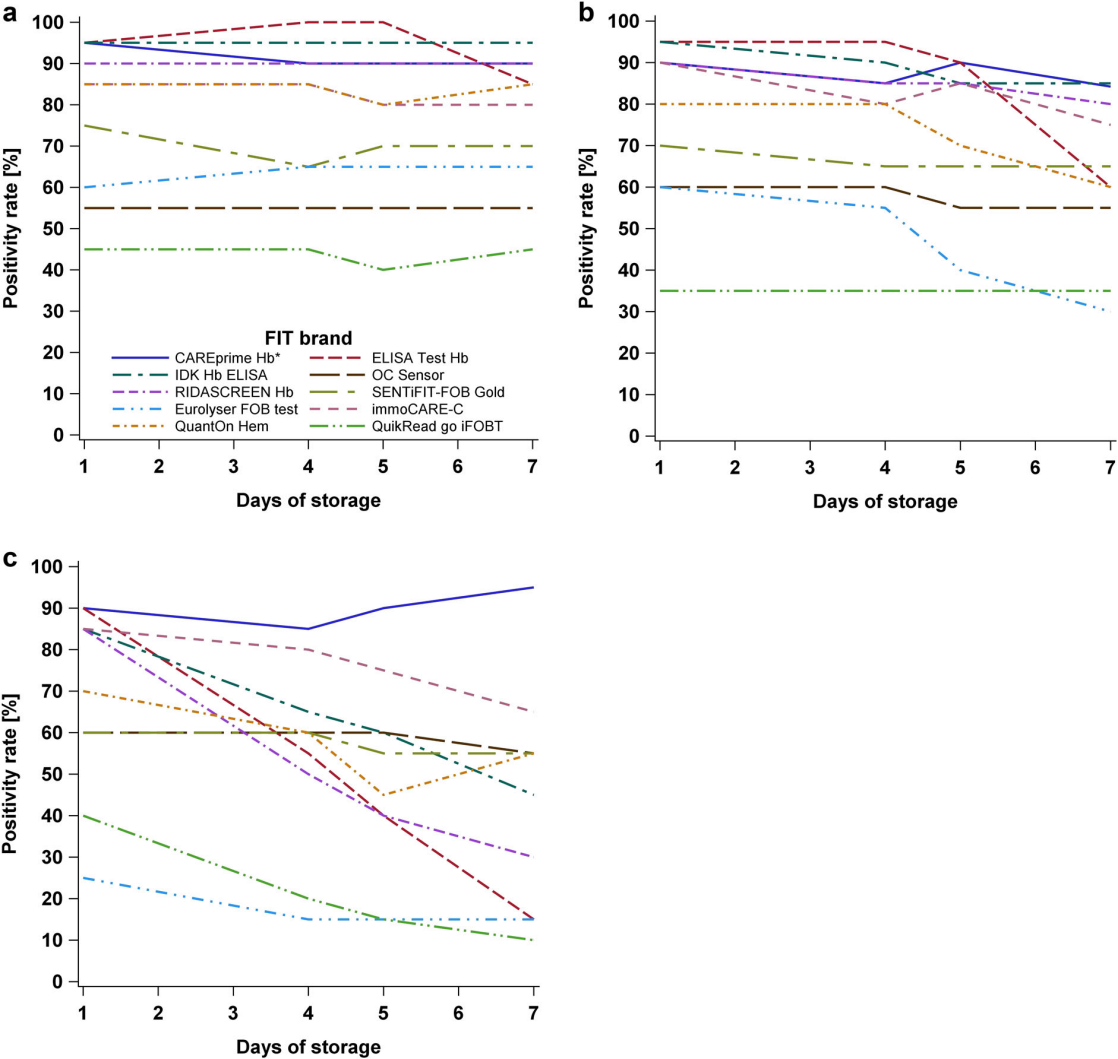
Positivity rate at preset manufacturers' thresholds at **a** 5, **b** 20, and **c** 35 °C, respectively. Legend in (**a**) is also applicable to **b** and **c**. *The analysis of CAREprime is based on 19 samples on the seventh day at 20 °C storage, whereas all other analyses are based on all 20 samples

**Fig. 4:**
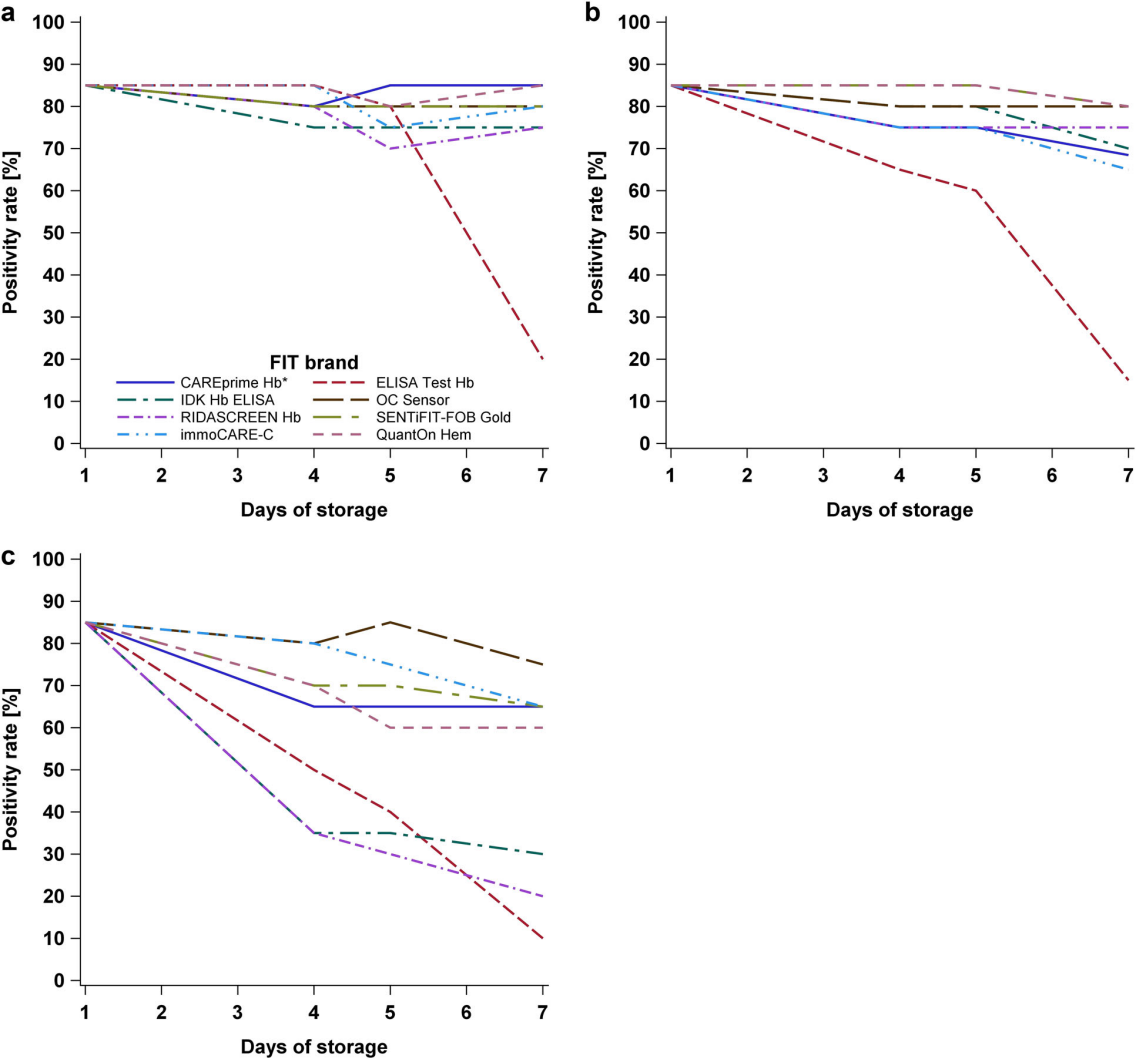
Positivity rate at adjusted thresholds yielding an equal positivity rate of 85% on the first day at **a** 5, **b** 20, and **c** 35 °C, respectively. Legend in (**a**) is also applicable to **b** and **c**. *The analysis of CAREprime is based on 19 samples on the seventh day at 20 °C storage, whereas all other analyses are based on all 20 samples.

Furthermore, the PR among all 20 study participants was computed and displayed (Fig. 3). Due to the large variation in threshold values preset by the manufacturers (range: 2–17 μg Hb/g feces), the latter analysis was repeated after adjusting the thresholds to yield the same PR on the first day for each test, in order to enhance comparability between the tests (Fig. 4). In addition, PRs with its exact 95% confidence interval (CI) were calculated, and differences in PRs at various combinations of temperature and storage time compared to 1-day storage at 5 °C were evaluated by McNemar test at preset and adjusted thresholds (Supplementary Table 3 and 4, respectively).

For one test (*CAREprime*), the analysis on day 7 at 20 °C storage temperature was based on 19 samples because of a missing test measurement, whereas all other analyses for *CAREprime* (and also for all other FITs) were based on the total sample size (*n* = 20).

Exact *p*-values were calculated and *p*-values of ≤0.05 were considered to be statistically significant. All statistical analyses were conducted using SAS Enterprise Guide, version 6.1 (SAS Institute Inc., Cary, North Carolina, USA).

## Results

### Study population

The 20 participants included in this analysis were recruited between 2006 and 2009. The mean age was 67 years (range: 56–80 years) and the majority of participants were males (60%). The most advanced finding at screening colonoscopy was advanced adenoma in five and non-advanced adenoma in eight cases. Two participants had hyperplastic polyps and five participants were without any findings at screening colonoscopy.

### Storage conditions

The median storage temperature (range) of the samples that were intended to be stored at 5, 20, and 35 °C was 6.1°C (8.4 °C–2.3 °C), 20.0 °C (17.8 °C–20.5 °C), and 34.7 °C (30.6 °C–35.2 °C), respectively. On the day of evaluation, the samples sent for laboratory analysis to the three locally located companies (Frost Diagnostika, Immundiagnostik, and R-Biopharm), arrived after a median delivery time of 1 h and 57 min, and the samples sent for laboratory analysis to CARE diagnostica arrived after a median delivery time of 3 h and 45 min. All samples were evaluated in parallel on the same day.

### Fecal Hb concentration

Figure 2 shows the fecal Hb concentration of ten quantitative FITs according to the different storage conditions. Based on the median Hb concentrations of individual FITs, the relative median Hb change (in percent) across all FITs on the fourth, fifth, and seventh day, respectively, in comparison to 1-day storing at 5 °C was calculated. One test (*QuikRead go iFOBT*) was excluded from this analysis because the median Hb concentration was already at the lower detection limit of 15 µg Hb/g feces and therefore no further decrease could be assessed:

- At 5 °C, the tests showed mostly fairly stable Hb concentrations, with constant median Hb levels and constant IQRs among all days of storage. The median change on the fourth, fifth, and seventh day was 0, 0, and only –5% units, respectively. For three tests (*ELISA Test Hb, IDK Hb ELISA,* and *SENTiFIT-FOB Gold*), statistically significant decrease in Hb concentration was observed (*p*-values < 0.05) (Supplementary Table 1).
- At 20 °C, the median change on the fourth, fifth, and seventh day went down by –21, –29, and –45% units, respectively, and six tests (*ELISA Test Hb, IDK Hb ELISA, SENTiFIT-FOB Gold, Eurolyser FOB test, immoCARE-C*, and *QuikRead go iFOBT*) indicated significant decrease in Hb concentration (*p*-values < 0.05) (Supplementary Table 1).
- At 35 °C, the median change was more pronounced at −78, −83, and −65% units on the fourth, fifth, and seventh day, respectively. For most FITs (*n* = 7), a significant decrease in Hb concentration (*p*-values < 0.05) was observed. For *ELISA Test Hb, IDK Hb ELISA, and Eurolyser FOB test*, a significant Hb decay in comparison to 5 °C was observable from day 1 on, and for *RIDASCREEN Hb* and *QuantOn Hem,* from day 4 on (Supplementary Table 1).

In summary, only two tests (CAREprime Hb and OC Sensor) showed stable Hb concentrations across all combinations of storage conditions in comparison to 1-day storing at 5 °C (*p*-values > 0.05) (Supplementary Table 1).

### Positivity rate at preset thresholds

Figure 3 show the PRs at preset thresholds (range: 2–17 μg Hb/g feces) recommended by the manufacturers according to the different storage conditions for all 20 study participants.

- At 5 °C, all tests presented fairly constant PRs, with changes of the PRs ranging from –10 to +5% units only among all days of storage.
- At 20 °C, the PR decreased for most of the tests only moderately with a prolonged storage time. The change of the PR on the fourth, fifth, and seventh day across the tests was up to −10, −20, and −35% units, respectively. For two tests (*ELISA Test Hb* and *Eurolyser FOB test*), the PR decreased significantly by ≥30% units until the seventh day (*p*-values < 0.05) (Supplementary Table 2).
- At 35 °C, substantial PR reduction over the following days was observed for several tests. The change of the PR on the fourth, fifth, and seventh day across the tests ranged up to −35, −50, and −75% units, respectively. For four tests (*ELISA Test Hb, IDK Hb ELISA, RIDASCREEN Hb*, and *Eurolyser FOB test*), the PRs went down significantly by ≥30% units from day 4 on, in comparison to 1-day storing at 5 °C (*p*-values < 0.05) (Supplementary Table 2).

In summary, four tests (*CAREprime Hb, OC Sensor, SENTiFIT-FOB Gold, and immoCARE-C*) showed constant PRs among all storage conditions, with differences in PR ranging from −20 to +5% units only (*p*-values > 0.05) (Supplementary Table 2).

### Positivity rate at adjusted thresholds

Figure 4 show the PRs at adjusted thresholds yielding the same PR of 85% (95% CI, 62–97%) (17 out of 20 samples) on day 1 at all three storage temperatures. The adjusted thresholds among the FITs ranged from 3 to 21 μg Hb/g feces, from 2 to 18 μg Hb/g feces, and from 2 to 11 μg Hb/g feces to set the same PR of 85% at 5, 20, and 35 °C, respectively. Two tests (*Eurolyser FOB test* and *QuikRead go iFOBT*) for which it was not possible to adjust the threshold to yield a PR of 85% because of their lower detection limits were not included in this analysis.

- At 5 °C, seven of the remaining eight tests showed fairly constant PRs (95% CI) ranging between 70% (46–88%) and 85% (62–97%) (between 75 and 85% in all but one instances) among all days of storage. For one test (*ELISA Test Hb*), the PR remained in the same range up to day 5, but significantly dropped to 20% (95% CI, 6–44%) at day 7 (*p*-value = 0.0002) (Supplementary Table 3).
- At 20 °C, the same seven tests still showed fairly constant PRs (95% CI) ranging between 75% (51–91%) and 85% (62–97%) up to day 5, and between 65% (41–85%) and 80% (56–94%) at day 7, whereas the PR of *ELISA Test Hb* significantly declined to 15% (95% CI, 3–38%) at day 7 (*p*-value = 0.0001) (Supplementary Table 3).
- At 35 °C, the PRs (95% CI) went down moderately to levels between 60% (36–81%) and 75% (51–91%) at day 7 for five of the tests (*OC Sensor, immoCARE-C, SENTiFIT-FOB Gold, CAREprime Hb, and QuantOn Hem*). However, for three tests (*IDK Hb ELISA, RIDASCREEN Hb*, and *ELISA test Hb*), the PRs (95% CI) declined significantly to values between 35 (15–59%) and 50% (27–73%) at day 4 and to PRs (95% CI) between 10 (1–32%) and 30% (12–54%) at day 7 (*p*-values < 0.05) (Supplementary Table 3).

## Discussion

To our knowledge, this is the first study performing a head-to-head comparison of a large number of quantitative FITs regarding the sample stability using exactly the same stool samples collected in a CRC screening setting. At cool storage temperature (here: 5 °C), almost all FITs showed fairly stable results over 7 days. At 20 °C, most FITs still showed fairly stable results over 4 days, whereas PRs significantly declined from day 4 on for most FITs at 35 °C. Although all FITs used special FSDs that were filled with a brand-specific preservative buffer to slow down any Hb decay, partly, very large differences between the FITs regarding the sample stability were observed.

In agreement with our results, several studies^21–24^ found a significant decrease of the PRs during summer, compared to winter with the *OC Sensor* (also included in our analysis). Although the stool-filled FSDs in these studies were partly kept in a refrigerator before analysis, higher ambient temperatures during the shipment were enough to reduce the fecal Hb concentration substantially. In a previous study from our group, Chen et al.^19^ investigated the *SENTiFIT-FOB Gold* (also included in our analysis) and found only a slight and non-significant reduction in the PR according to season of sample collection. Therefore it was essentially unclear whether and to what extent the differences between different FIT brands regarding the sample stability existed.

In another study also investigating the *OC Sensor*, van Rossum et al.^25^ found a substantial reduction of the PR, leading to reduced detection rates with a prolonged sample return time. By contrast, a study led by van Roon et al.^22^ did not observe any substantial reduction in the PR for the *OC Sensor*, with a prolonged sample return time. Because in these studies, the stool-filled FSDs were also partly kept in a refrigerator; it remained unclear to what extent prolonged storing at higher temperatures had an impact on the PR and if this is also transferable to other FIT brands.

In a study from France, Guittet et al.^26^ directly compared three different FITs (*OC Sensor, FOB Gold,* and *Magstream HT*) and found a superior Hb stabilization ability for *OC Sensor* in comparison to the two other FITs. However, because the fecal samples from ten initially FIT-negative, healthy and young volunteers were artificially spiked with human blood; it was unclear to what extent these results are transferable to “real life” CRC screening participants, including patients with colorectal neoplasms. In our study, we used samples from 20 participants of screening colonoscopy including participants with advanced and non-advanced adenomas and found similar Hb stabilities between two of these FITs (*OC Sensor* and *SENTiFIT-FOB Gold*). In agreement with our findings, Guittet et al found relatively stable fecal Hb concentrations among all three FITs at cool storage temperatures, but a substantial decrease already at temperatures above 20 °C and also with prolonged storage times.

Our study has specific strengths which include the parallel evaluation of a large number of quantitative FITs using exactly the same stool samples of CRC screening participants. The study design essentially precluded any differences in study populations or sample handling as a cause of observed differences for sample stabilization between tests. All FITs were evaluated in exactly the same study participants who were recruited in a CRC screening setting among participants of screening colonoscopy. Stool samples were collected in exactly the same manner, and additional homogenization of the stool samples after thawing and prior to sample extraction for the individual FITs should further have eliminated any variation of fecal Hb concentrations within a single bowel movement. Furthermore, all collected stool specimens, using the brand-specific FSD, were stored in parallel under identical storage conditions (temperature and time) until few hours (during shipment) before conducting the FIT measurements on the respective days.

However, our study also has limitations. Stool samples were originally collected in stool containers (60 ml) rather than with the FSDs provided by the manufacturers and stored frozen at −80 °C over several years prior to analysis. Our study design though may have been the only way to conduct a parallel Hb stabilization study like this, as it is hard to imagine that screening participants would be willing and able to fill in parallel three FSDs for each of ten different FIT brands (in total, n=30), each with different sample collection instructions. Furthermore, it would be almost impossible to ensure exactly the same temperature conditions for all FITs if not performing all the preparation steps at the same place. Although not used for the initial stool collection by the participants, the original FSDs provided by the manufacturers were used when extracting a defined amount of stool from the thawed stool samples, and the samples were stored in their respective preservative buffer systems during the study. Furthermore, the stool-filled FSDs were homogenized on a vortexer so that the stool could completely disperse into the buffer to ensure optimal Hb stabilization in the buffer. Following the manufacturers' instructions, the stool-filled FSDs were stored overnight in their original FSDs to enable a sufficiently long time period for any Hb to move out of the stool into the preservative buffer. Additional homogenization on a vortexer on the next day, just before aliquoting the total volume into the four safe-lock tubes, should have ruled out any variation of Hb concentration within the same FSD. Another limitation is that the number of stool samples (*n* = 20) was rather low, leading to broad IQRs and 95% CIs, but despite that statistical differences between FITs regarding the sample stability were observed. However, to obtain 20 FIT-positive samples within a fecal Hb concentration range for which a potential Hb decay may turn a positive to a negative test result and therefore be highly clinically relevant, several hundred participants of screening colonoscopy are necessary to recruit. In theory, about 400 participants would be necessary to recruit, with each participant asked to collect three FSDs from ten FITs (30 sampling tubes in total, which is almost impossible to imagine), of which “only” 20 FIT-positive stool samples (assuming a PR of 5%) would be included in the final stability analysis. Furthermore, despite the limited number of study participants finally included, this study is based on a total number of 20 × 10 (tests) × 4 (days) × 3 (temperatures) = 2400 FITs, and, to the best of our knowledge, the first and only one that provides a comparative evaluation of FIT stability across multiple FITs under fully comparable conditions.

Our analysis was restricted to storage at constant temperatures and to fecal samples with an initial Hb concentration (measured in a previous analysis by one specific FIT) between 10 and 100 µg Hb/g feces. In most settings, the temperature is likely to vary over time, and constant temperatures around 35 °C or even 20 °C would not be expected in higher latitude countries, not even in summer. Therefore the Hb decay and the proportion of false-negative findings due to Hb decay would be expected to be lower in real settings in such countries than observed in our study. Also, false-negative results would be highly unlikely in rare cases of initial Hb concentration > 100 µg Hb/g feces which were not included in our study, but are typical among CRC patients^27^.

Despite its limitations, our study provides important information regarding sample Hb stability and its comparability for a large number of quantitative FITs that are increasingly used in CRC screening practice. A constant cool storage of stool-filled FSDs from sampling until analysis was shown to result in fairly constant PRs and fecal Hb concentrations throughout 7 days of observation. At 20 °C, most FITs still showed fairly stable results over 4 days, whereas PRs significantly declined from day 4 on for most FITs at 35 °C. To achieve constant PRs, which reflect the consequent colonoscopy workload, constant detection rates screening programs should aim at minimizing Hb decay by either keeping stool-filled FSDs in a cooled environment from sampling until evaluation, limiting the time period between sampling and test evaluation and/or selecting a FIT brand that combines good diagnostic performance with maximum possible stability even at prolonged sample return time at higher temperature. We hope that our results may help in guiding the design and monitoring of FIT-based screening programs and to stimulate further efforts to optimize FIT buffers in order to enhance and maximize Hb stability.

## Study Highlights

### WHAT IS CURRENT KNOWLEDGE

- A large number of fecal immunochemical tests (FITs) are meanwhile being offered for colorectal cancer screening.
- A decrease in FIT positivity rates at higher ambient temperatures has been reported, but it is unclear to what extent different FIT brands are affected.

### WHAT IS NEW HERE

- Ten different FITs were directly compared using stool samples from true screening participants.
- At 5 °C, almost all FITs showed fairly stable results throughout the 7-day observation period. At 20 °C, most FITs still showed fairly stable results over 4 days, whereas positivity rates significantly declined from day 4 on for most FITs at 35 °C.
- Major differences regarding the sample stability between FITs were observed.
- FIT-specific sample stability according to ambient temperature and time period between sampling and test evaluation requires careful consideration in FIT-based screening programs.

## Electronic supplementary material

Supplementary Tables
