# Supplementary material for: Direct comparison of ten quantitative fecal immunochemical tests for hemoglobin stability in colorectal cancer screening
Source: Clin Transl Gastroenterol. 2018 Jul 6;9(7):168. doi: 10.1038/s41424-018-0035-2 (PMC6033915; doi:10.1038/s41424-018-0035-2)
Supplement: Supplementary file 1 — Supplementary Tables [file 41424_2018_35_MOESM1_ESM.docx]

**Supplementary Table 1** Median fecal hemoglobin concentration [µg Hb/g feces] according to FIT brand and storage condition

| **FIT brand** | **Storage**  **Temperature** | **Storage day 1** | | **Storage day 4** | | **Storage day 5** | | **Storage day 7** | |
| --- | --- | --- | --- | --- | --- | --- | --- | --- | --- |
|  |  | **Median (IQR)** | **p-value** | **Median (IQR)** | **p-value** | **Median (IQR)** | **p-value** | **Median (IQR)** | **p-value** |
| CAREprime Hb | 5° C | 27 (10-58) | Reference | 28 (11-57) | 0.2058 | 28 (11-56) | 0.2651 | 30 (11-59) | 0.5521 |
|  | 20° C | 28 (11-66) | 0.6150 | 29 (11-61) | 0.4749 | 28 (11-61) | 0.4304 | 27 (9-57)* | 0.1206 |
|  | 35° C | 35 (13-63) | 0.5459 | 26 (10-45) | 0.4304 | 25 (9-51) | 0.5706 | 23 (8-47) | 0.4144 |
| ELISA Test Hb | 5° C | 21 (21-21) | Reference | 21 (21-21) | 0.8750 | 21 (21-21) | 0.6250 | 4 (3-13) | **<.0001** |
|  | 20° C | 21 (21-21) | 0.8438 | 21 (13-21) | 0.3125 | 21 (7-21) | 0.0840 | 2 (1-7) | **<.0001** |
|  | 35° C | 16 (6-21) | **0.0098** | 2 (1-12) | **<.0001** | 1 (0-9) | **<.0001** | 1 (0-1) | **<.0001** |
| IDK Hb ELISA | 5° C | 29 (10-44) | Reference | 23 (8-37) | 0.0539 | 23 (9-38) | **0.0230** | 22 (9-37) | **0.0385** |
|  | 20° C | 26 (8-36) | 0.1324 | 14 (5-23) | **0.0004** | 11 (5-23) | **0.0082** | 10 (3-21) | **0.0001** |
|  | 35° C | 10 (5-20) | **0.0172** | 3 (1-6) | **<.0001** | 3 (1-5) | **<.0001** | 2 (1-4) | **<.0001** |
| OC Sensor | 5° C | 14 (4-28) | Reference | 15 (4-32) | 0.9295 | 16 (5-32) | 0.9843 | 16 (5-32) | 0.3683 |
|  | 20° C | 15 (4-27) | 0.6722 | 16 (3-49) | 0.3038 | 16 (4-26) | 0.5798 | 16 (3-26) | 0.1779 |
|  | 35° C | 14 (4-25) | 0.6944 | 13 (5-22) | 0.6226 | 13 (5-22) | 0.6022 | 13 (4-21) | 0.5872 |
| RIDASCREEN Hb | 5° C | 50 (21-50) | Reference | 50 (19-50) | 0.6377 | 50 (15-50) | 0.4648 | 50 (16-50) | 0.4697 |
|  | 20° C | 50 (15-50) | 0.7695 | 49 (12-50) | 0.2490 | 42 (11-50) | 0.2661 | 27 (10-50) | 0.0923 |
|  | 35° C | 33 (12-50) | 0.1909 | 8 (4-27) | **0.0002** | 5 (4-14) | **<.0001** | 5 (3-9) | **<.0001** |
| SENTiFIT-FOB Gold | 5° C | 60 (17-86) | Reference | 50 (10-87) | **0.0295** | 52 (10-87) | **0.0083** | 50 (10-87) | **0.0225** |
|  | 20° C | 47 (6-91) | **0.0203** | 46 (6-91) | **0.0107** | 43 (5-87) | **0.0052** | 39 (5-55) | **0.0001** |
|  | 35° C | 45 (8-107) | 0.1237 | 28 (2-59) | **<.0001** | 25 (2-51) | **<.0001** | 30 (2-82) | **0.0004** |
| Eurolyser FOB test | 5° C | 15 (4-32) | Reference | 15 (4-34) | 0.5171 | 16 (3-32) | 0.8176 | 15 (4-29) | 0.7819 |
|  | 20° C | 12 (3-30) | 0.1297 | 8 (2-23) | **0.0017** | 7 (2-21) | **0.0002** | 5 (2-14) | **0.0002** |
|  | 35° C | 3 (2-10) | **0.0267** | 2 (2-3) | **0.0066** | 2 (2-2) | **0.0038** | 2 (2-3) | **0.0056** |
| immoCARE-C | 5° C | 30 (14-67) | Reference | 27 (15-55) | 0.7381 | 22 (10-50) | 0.1893 | 28 (13-56) | 0.1231 |
|  | 20° C | 25 (11-49) | 0.7562 | 24 (13-24) | 0.1536 | 21 (9-41) | **0.0400** | 21 (7-36) | **0.0136** |
|  | 35° C | 19 (10-39) | 0.2493 | 18 (8-37) | 0.1769 | 17 (6-28) | 0.0759 | 11 (4-27) | **0.0484** |
| QuantOn Hem | 5° C | 19 (6-25) | Reference | 17 (9-21) | 0.8124 | 16 (7-23) | 0.6742 | 16 (9-20) | 0.7285 |
|  | 20° C | 15 (7-22) | 0.6742 | 13 (5-19) | 0.2024 | 11 (3-17) | 0.0583 | 10 (3-18) | 0.1536 |
|  | 35° C | 11 (3-20) | 0.2455 | 4 (2-13) | **0.0153** | 3 (1-13) | **0.0039** | 7 (1-10) | **0.0192** |
| QuikRead go iFOBT | 5° C | 15 (15-36) | Reference | 15 (15-32) | 0.5181 | 15 (15-31) | 0.3057 | 15 (15-29) | 0.2441 |
|  | 20° C | 15 (15-34) | 0.0977 | 15 (15-28) | 0.0898 | 15 (15-26) | 0.1343 | 15 (15-25) | **0.0273** |
|  | 35° C | 15 (15-23) | 0.6477 | 15 (15-15) | 0.3008 | 15 (15-15) | 0.1602 | 15 (15-15) | 0.0879 |

FIT=Fecal immunochemical test; IQR=Interquartile range; Hb=Hemoglobin; bold type p-value indicates significant difference in fecal Hb concentration compared to one-day storing at 5°C; *Analysis based on 19 samples

**Supplementary Table 2** Positivity rate [%] at preset thresholds according to FIT brand and storage condition

| **FIT brand** | **Storage**  **Temperature** | **Storage day 1** | | **Storage day 4** | | **Storage day 5** | | **Storage day 7** | |
| --- | --- | --- | --- | --- | --- | --- | --- | --- | --- |
|  |  | **PR (95% CI)** | **p-value** | **PR (95% CI)** | **p-value** | **PR (95% CI)** | **p-value** | **PR (95% CI)** | **p-value** |
| CAREprime Hb | 5° C | 95 (75-100) | Reference | 90 (68-99) | 1.0000 | 90 (68-99) | 1.0000 | 90 (68-99) | 1.0000 |
|  | 20° C | 90 (68-99) | 1.0000 | 85 (62-97) | 0.5000 | 90 (68-99) | 1.0000 | 84 (60-97)* | 0.5000 |
|  | 35° C | 90 (68-99) | 1.0000 | 85 (62-97) | 0.6250 | 90 (68-99) | 1.0000 | 95 (75-100) | 1.0000 |
| ELISA Test Hb | 5° C | 95 (75-100) | Reference | 100 (83-100) | 1.0000 | 100 (83-100) | 1.0000 | 85 (62-97) | 0.6250 |
|  | 20° C | 95 (75-100) | 1.0000 | 95 (75-100) | 1.0000 | 90 (68-99) | 1.0000 | 60 (36-81) | **0.0391** |
|  | 35° C | 90 (68-99) | 1.0000 | 55 (32-77) | **0.0078** | 40 (19-64) | **0.0010** | 15 (3-38) | **<.0001** |
| IDK Hb ELISA | 5° C | 95 (75-100) | Reference | 95 (75-100) | 1.0000 | 95 (75-100) | 1.0000 | 95 (75-100) | 1.0000 |
|  | 20° C | 95 (75-100) | 1.0000 | 90 (68-99) | 1.0000 | 85 (62-97) | 0.5000 | 85 (62-97) | 0.5000 |
|  | 35° C | 85 (62-97) | 0.5000 | 65 (41-85) | **0.0313** | 60 (36-81) | **0.0156** | 40 (19-64) | **0.0020** |
| OC Sensor | 5° C | 55 (32-77) | Reference | 55 (32-77) | 1.0000 | 55 (32-77) | 1.0000 | 55 (32-77) | 1.0000 |
|  | 20° C | 60 (36-81) | 1.0000 | 60 (36-81) | 1.0000 | 55 (32-77) | 1.0000 | 55 (32-77) | 1.0000 |
|  | 35° C | 60 (36-81) | 1.0000 | 60 (36-81) | 1.0000 | 60 (36-81) | 1.0000 | 55 (32-77) | 1.0000 |
| RIDASCREEN Hb | 5° C | 90 (68-99) | Reference | 90 (68-99) | 1.0000 | 90 (68-99) | 1.0000 | 90 (68-99) | 1.0000 |
|  | 20° C | 90 (68-99) | 1.0000 | 85 (62-97) | 1.0000 | 85 (62-97) | 1.0000 | 80 (56-94) | 0.6250 |
|  | 35° C | 85 (62-97) | 1.0000 | 50 (27-73) | **0.0078** | 40 (19-64) | **0.0020** | 30 (12-54) | **0.0005** |
| SENTiFIT-FOB Gold | 5° C | 75 (51-91) | Reference | 65 (41-85) | 0.5000 | 70 (46-88) | 1.0000 | 70 (46-88) | 1.0000 |
|  | 20° C | 70 (46-88) | 1.0000 | 65 (41-85) | 0.5000 | 65 (41-85) | 0.5000 | 65 (41-85) | 0.5000 |
|  | 35° C | 60 (36-81) | 0.2500 | 60 (36-81) | 0.2500 | 55 (32-77) | 0.1250 | 55 (32-77) | 0.1250 |
| Eurolyser FOB test | 5° C | 60 (36-81) | Reference | 65 (41-85) | 1.0000 | 65 (41-85) | 1.0000 | 65 (41-85) | 1.0000 |
|  | 20° C | 60 (36-81) | 1.0000 | 55 (32-77) | 1.0000 | 40 (19-64) | 0.2188 | 30 (12-54) | **0.0313** |
|  | 35° C | 25 (9-49) | 0.0654 | 15 (3-38) | **0.0225** | 15 (3-38) | **0.0117** | 15 (3-38) | **0.0225** |
| immoCARE-C | 5° C | 85 (62-97) | Reference | 85 (62-97) | 1.0000 | 80 (56-94) | 1.0000 | 80 (56-94) | 1.0000 |
|  | 20° C | 90 (68-99) | 1.0000 | 80 (56-94) | 1.0000 | 85 (62-97) | 1.0000 | 75 (51-91) | 0.6250 |
|  | 35° C | 85 (62-97) | 1.0000 | 80 (56-94) | 1.0000 | 75 (51-91) | 0.7266 | 65 (41-85) | 0.2891 |
| QuantOn Hem | 5° C | 85 (62-97) | Reference | 85 (62-97) | 1.0000 | 80 (56-94) | 1.0000 | 85 (62-97) | 1.0000 |
|  | 20° C | 80 (56-94) | 1.0000 | 80 (56-94) | 1.0000 | 70 (46-88) | 0.3750 | 60 (36-81) | 0.1250 |
|  | 35° C | 70 (46-88) | 0.3750 | 60 (36-81) | 0.1250 | 45 (23-68) | **0.0215** | 55 (32-77) | 0.1094 |
| QuikRead go iFOBT | 5° C | 45 (23-68) | Reference | 45 (23-68) | 1.0000 | 40 (19-64) | 1.0000 | 45 (23-68) | 1.0000 |
|  | 20° C | 35 (15-59) | 0.6250 | 35 (15-59) | 0.7266 | 35 (15-59) | 0.7266 | 35 (15-59) | 0.6250 |
|  | 35° C | 40 (19-64) | 1.0000 | 20 (6-44) | 0.0625 | 15 (3-38) | 0.0703 | 10 (1-32) | **0.0391** |

FIT=Fecal immunochemical test; CI=Confidence interval; Hb=Hemoglobin; PR=Positivity rate; bold type p-value indicates significant difference in positivity rate compared to one-day storing at 5°C; *Analysis based on 19 samples

**Supplementary Table 3** Positivity rate [%] at adjusted thresholds according to FIT brand and storage condition

| **FIT brand** | **Storage**  **Temperature** | **Storage day 1** | | **Storage day 4** | | **Storage day 5** | | **Storage day 7** | |
| --- | --- | --- | --- | --- | --- | --- | --- | --- | --- |
|  |  | **PR (95% CI)** | **p-value** | **PR (95% CI)** | **p-value** | **PR (95% CI)** | **p-value** | **PR (95% CI)** | **p-value** |
| CAREprime Hb | 5° C | 85 (62-97) | Reference | 80 (56-94) | 1.0000 | 85 (62-97) | 1.0000 | 85 (62-97) | 1.0000 |
|  | 20° C | 85 (62-97) | 1.0000 | 75 (51-91) | 0.6250 | 75 (51-91) | 0.6250 | 68 (43-87)* | 0.3750 |
|  | 35° C | 85 (62-97) | 1.0000 | 65 (41-85) | 0.2891 | 65 (41-85) | 0.2891 | 65 (41-85) | 0.2891 |
| ELISA Test Hb | 5° C | 85 (62-97) | Reference | 85 (62-97) | 1.0000 | 80 (56-94) | 1.0000 | 20 (6-44) | **0.0002** |
|  | 20° C | 85 (62-97) | 1.0000 | 65 (41-85) | 0.2188 | 60 (36-81) | 0.1250 | 15 (3-38) | **0.0001** |
|  | 35° C | 85 (62-97) | 1.0000 | 50 (27-73) | **0.0391** | 40 (19-64) | **0.0039** | 10 (1-32) | **<.0001** |
| IDK Hb ELISA | 5° C | 85 (62-97) | Reference | 75 (51-91) | 0.6250 | 75 (51-91) | 0.6250 | 75 (51-91) | 0.6250 |
|  | 20° C | 85 (62-97) | 1.0000 | 80 (56-94) | 1.0000 | 80 (56-94) | 1.0000 | 70 (46-88) | 0.3750 |
|  | 35° C | 85 (62-97) | 1.0000 | 35 (15-59) | **0.0020** | 35 (15-59) | **0.0063** | 30 (12-54) | **0.0010** |
| OC Sensor | 5° C | 85 (62-97) | Reference | 80 (56-94) | 1.0000 | 80 (56-94) | 1.0000 | 80 (56-94) | 1.0000 |
|  | 20° C | 85 (62-97) | 1.0000 | 80 (56-94) | 1.0000 | 80 (56-94) | 1.0000 | 80 (56-94) | 1.0000 |
|  | 35° C | 85 (62-97) | 1.0000 | 80 (56-94) | 1.0000 | 85 (62-97) | 1.0000 | 75 (51-91) | 0.7266 |
| RIDASCREEN Hb | 5° C | 85 (62-97) | Reference | 80 (56-94) | 1.0000 | 70 (46-88) | 0.2500 | 75 (51-91) | 0.6250 |
|  | 20° C | 85 (62-97) | 1.0000 | 75 (51-91) | 0.6250 | 75 (51-91) | 0.6250 | 75 (51-91) | 0.6250 |
|  | 35° C | 85 (62-97) | 1.0000 | 35 (15-59) | **0.0063** | 30 (12-54) | **0.0034** | 20 (6-44) | **0.0002** |
| SENTiFIT-FOB Gold | 5° C | 85 (62-97) | Reference | 80 (56-94) | 1.0000 | 80 (56-94) | 1.0000 | 80 (56-94) | 1.0000 |
|  | 20° C | 85 (62-97) | 1.0000 | 85 (62-97) | 1.0000 | 85 (62-97) | 1.0000 | 80 (56-94) | 1.0000 |
|  | 35° C | 85 (62-97) | 1.0000 | 70 (46-88) | 0.2500 | 70 (46-88) | 0.2500 | 65 (41-85) | 0.1250 |
| immoCARE-C | 5° C | 85 (62-97) | Reference | 85 (62-97) | 1.0000 | 75 (51-91) | 0.5000 | 80 (56-94) | 1.0000 |
|  | 20° C | 85 (62-97) | 1.0000 | 75 (51-91) | 0.6250 | 75 (51-91) | 0.6250 | 65 (41-85) | 0.2188 |
|  | 35° C | 85 (62-97) | 1.0000 | 80 (56-94) | 1.0000 | 75 (51-91) | 0.7266 | 65 (41-85) | 0.2891 |
| QuantOn Hem | 5° C | 85 (62-97) | Reference | 85 (62-97) | 1.0000 | 80 (56-94) | 1.0000 | 85 (62-97) | 1.0000 |
|  | 20° C | 85 (62-97) | 1.0000 | 85 (62-97) | 1.0000 | 85 (62-97) | 1.0000 | 80 (56-94) | 1.0000 |
|  | 35° C | 85 (62-97) | 1.0000 | 70 (46-88) | 0.3750 | 60 (36-81) | 0.1250 | 60 (36-81) | 0.1797 |

FIT=Fecal immunochemical test; CI=Confidence interval; Hb=Hemoglobin; PR=Positivity rate; bold type p-value indicates significant difference in positivity rate compared to one-day storing at 5°C; * Analysis based on 19 samples
